# Implementation of the World Health Organization Minimum Dataset for Emergency Medical Teams to Create Disaster Profiles for the Indonesian SATUSEHAT Platform Using Fast Healthcare Interoperability Resources: Development and Validation Study

**DOI:** 10.2196/59651

**Published:** 2024-08-28

**Authors:** Hiro Putra Faisal, Masaharu Nakayama

**Affiliations:** 1 Department of Medical Informatics, Tohoku University Graduate School of Medicine, 2-1 Seiryo-machi, Aoba-ku, Sendai, 980-8574, Japan, 81 22-717-7572, 81 22-717-7505; 2 Department of Physiology, Faculty of Medicine, UIN Syarif Hidayatullah Jakarta, Tangerang Selatan, Indonesia

**Keywords:** WHO EMT MDS, FHIR, SATUSEHAT, disaster, implementation, development, validation, emergency medical team, disaster management, Indonesia, Fast Healthcare Interoperability Resources, resources, interoperability, electronic medical records, EMR, reporting, disaster profile, health data, health data collection, World Health Organization, EMT, WHO, MDS, minimum dataset

## Abstract

**Background:**

The National Disaster Management Agency (*Badan Nasional Penanggulangan Bencana*) handles disaster management in Indonesia as a health cluster by collecting, storing, and reporting information on the state of survivors and their health from various sources during disasters. Data were collected on paper and transferred to Microsoft Excel spreadsheets. These activities are challenging because there are no standards for data collection. The World Health Organization (WHO) introduced a standard for health data collection during disasters for emergency medical teams (EMTs) in the form of a minimum dataset (MDS). Meanwhile, the Ministry of Health of Indonesia launched the SATUSEHAT platform to integrate all electronic medical records in Indonesia based on Fast Healthcare Interoperability Resources (FHIR).

**Objective:**

This study aims to implement the WHO EMT MDS to create a disaster profile for the SATUSEHAT platform using FHIR.

**Methods:**

We extracted variables from 2 EMT MDS medical records—the WHO and Association of Southeast Asian Nations (ASEAN) versions—and the daily reporting form. We then performed a mapping process to match these variables with the FHIR resources and analyzed the gaps between the variables and base resources. Next, we conducted profiling to see if there were any changes in the selected resources and created extensions to fill the gap using the Forge application. Subsequently, the profile was implemented using an open-source FHIR server.

**Results:**

The total numbers of variables extracted from the WHO EMT MDS, ASEAN EMT MDS, and daily reporting forms were 30, 32, and 46, with the percentage of variables matching FHIR resources being 100% (30/30), 97% (31/32), and 85% (39/46), respectively. From the 40 resources available in the FHIR ID core, we used 10, 14, and 9 for the WHO EMT MDS, ASEAN EMT MDS, and daily reporting form, respectively. Based on the gap analysis, we found 4 variables in the daily reporting form that were not covered by the resources. Thus, we created extensions to address this gap.

**Conclusions:**

We successfully created a disaster profile that can be used as a disaster case for the SATUSEHAT platform. This profile may standardize health data collection during disasters.

## Introduction

In Indonesia, disaster management is conducted by the National Disaster Management Agency (*Badan Nasional Penanggulangan Bencana*) [1]. Specifically, health issues during disasters are mandated to health clusters, whose members consist of regional health services, rapid health assessment teams, and emergency medical teams (EMTs) from various institutions. The health cluster collects, records, and reports information on survivors and their health conditions during a disaster [2]. According to the World Health Organization (WHO), collecting patient data requires the use of a nationally accepted reporting form or an approved dataset that is reported periodically. This report must include copies for the patient [3]. The current standard form available in Indonesia is the Rapid Health Assessment Form established by the Ministry of Health (MoH). This form summarized only the number of survivors and their general situation [2]. Meanwhile, the EMTs from various institutions recorded the survivors’ health status using their forms [4]. This information was collected by *Badan Nasional Penanggulangan Bencana* during daily reporting meetings and transferred to Microsoft Excel spreadsheets [5]. This activity is time-consuming and often inaccurate [6]. Additionally, the lack of coordination due to the decreased number of officers affected by the disaster may have impacted data collection and information exchange [7]. Consequently, this affects the handling of survivors at disaster locations and directs them to nearby health facilities.

Several institutions have tried to develop applications to record medical data [8], construct a documentation form [9-11], and create the minimum dataset (MDS) needed during disasters [11,12]. However, none of these are available for general use. To address this problem, the WHO introduced an MDS for EMTs in 2017 for use during disaster events [12]. This form has been tested and used to assess several disasters worldwide [13]. In Southeast Asian countries, the EMT MDS was introduced through the Project for Strengthening the Association of Southeast Asian Nations (ASEAN) Regional Capacity on Disaster Health Management (ARCH Project) [14]. The ARCH Project aimed to strengthen disaster management for ASEAN members through collaboration with the Japan International Cooperation Agency. One of the main goals is to use the WHO EMT MDS standards in the ASEAN region.

Indonesia’s MoH launched the SATUSEHAT platform based on Fast Healthcare Interoperability Resources (FHIR) [15]. This action aims to integrate and perform interoperability among health care facilities. Thus, the government has targeted all health care facilities to have electronic medical records by the end of 2023 and be interoperable using this platform [16].

FHIR is a standard for exchanging health care information through health information systems developed by the health care standards organization Health Level Seven International [17]. FHIR uses a representational state-transfer (REST) application programming interface (API), a common web service architecture that aims to make it easier for health care systems to share and access data, eventually improving the interoperability of health care information. The main features of FHIR are modularity, standardized resources, and interoperability, thus making it easy to work with specific data elements such as patient demographics, health conditions, and medications with different health care systems.

With this momentum, we conducted this research to map the WHO EMT MDS form to FHIR so that it can be integrated into the SATUSEHAT platform. This will help survivors of disasters to record, report, and refer to systems.

## Methods

### Extract Variables From the Medical Records

The EMT MDS consists of several forms that must be filled out during disasters. The set includes a medical records form combined with a tick-box section and the daily reporting form, both of which are sent to the EMT Coordination Cell (EMTCC). In the medical records section and tick-box form, EMT officers fill in patient data such as identity, medical history, vital signs, physical examination, and therapy management, which must be signed by a doctor. In this study, the tick box only helps to highlight the patient’s condition, which is divided into age, sex, health events, procedures and outcomes, and context, without the patient’s identity to maintain anonymity. Afterward, the form will be collected by the EMTCC to aggregate the data into the daily reporting form. The daily reporting form is part of the EMT MDS designed by the WHO to be used in the EMTCC office. The daily reporting form itself has already been tested during disasters in some countries [13,18,19]. It contains basic information regarding the team, the location to which the team was assigned, daily summaries of the facility, accumulation of data from the tick boxes as MDS statistics, and information regarding crucial needs and risks [20].

**Figure 1.**
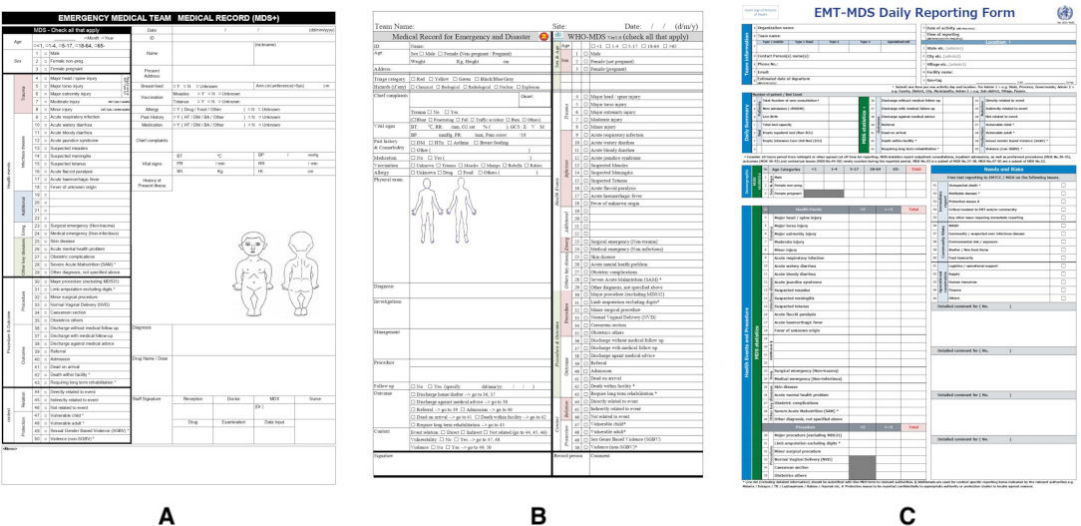
Form of medical records taken from the (A) WHO EMT MDS, (B) ASEAN EMT MDS, and (C) daily reporting form. ASEAN: Association of Southeast Asian Nations; EMT: emergency medical team; MDS: minimum dataset; WHO: World Health Organization.

This study extracted data elements from the medical records form and daily reporting form. We obtained 2 variations of medical records from the WHO EMT MDS and the ASEAN EMT MDS, which are adaptations for ASEAN countries developed by the ARCH Project. These forms are illustrated in Figure 1.

We chose this medical record form for Indonesia since the Indonesian government, as a member of ASEAN states, has already been trained to use the EMT MDS during regional collaboration drills on the ARCH Project [21,22]. This project aims to strengthen disaster health management in ASEAN countries and meet the WHO EMT MDS as a standard operational procedure [23]. Thus, digitizing this set will be an appropriate choice.

### Mapping and Gap Analysis

Next, we manually mapped these data elements onto the base resources. Based on the results, we conducted a gap analysis between the data elements and FHIR resources. We divided the EMT MDS elements into the medical record and daily reporting form, including the tick-box section. Next, we examined whether each data element and the FHIR base resources matched.

### Profiling and Validation

Profiling is the process of defining the profile created by setting the cardinality and creating an extension to the data elements that have definitions in the resource base. We used the Forge application since it is a common tool used in profiling research [24,25]. Next, we validated the profiles using the SIMPLIFIER.NET website [26].

### Implementation

We implemented the profiles in the HAPI framework using an open-source FHIR server [27]. We used the Insomnia application, an open-source application that performs the API testing and development process, to perform the POST request to the server with body content using JSON [28].

### Ethical Considerations

Since our research did not involve human subjects and no actual patient data were included in this study, ethics approval was not required.

## Results

### Mapping

We created a list and group of variables extracted from the WHO and ASEAN EMT MDS medical records. The data were divided into 2 categories: medical and daily reporting forms. Subsequently, the base FHIR was mapped. The list of variables and mapping process results from the WHO EMT MDS and ASEAN EMT MDS medical records forms are shown in Table 1. Meanwhile, Table 2 shows the variables in the daily reporting form mapped to the FHIR resources.

From the 40 resources available in the FHIR ID core, we selected 10, 14, and 9 resources for the WHO EMT MDS, ASEAN EMT MDS, and daily reporting form, respectively, as displayed in Table 3. The total numbers of variables from the WHO EMT MDS medical record form, the ASEAN EMT MDS medical record form, and the daily reporting form with variables that matched the FHIR resources are listed in Table 4.

**Table 1.** Mapping results for the medical forms.

Variable		WHO^a^ EMT^b^ MDS^c^	ASEAN^d^ EMT MDS	FHIR^e^ resources
Team name		N/A^f^	✓^g^	Organization
Site		N/A	✓	Location
Date		✓	✓	Encounter
ID		✓	✓	Patient
Name		✓	✓	Patient
Age		N/A	╳^h^	N/A
Nickname		✓	N/A	Patient
Present address		✓	✓	Patient
Triage category		N/A	✓	Observation
Hazards (if any)		N/A	✓	Observation
Breastfeeding		✓	✓	Observation
Arm circumference (<5 y)		✓	N/A	Observation
Vaccination		✓	✓	Immunization
Allergy		✓	✓	AllergyIntolerance
Past history		✓	✓	Condition
Medication		✓	✓	MedicationStatement
Chief complaints		✓	✓	Observation
Onset		N/A	✓	Condition
Trauma		N/A	✓	Observation
**Vital signs**				
	BT^i^	✓	✓	Observation
	PR^j^	✓	✓	Observation
	BP^k^	✓	✓	Observation
	RR^l^	✓	✓	Observation
	O_2_ sat^m^	N/A	✓	Observation
	GCS^n^	N/A	✓	Observation
	Pain score	N/A	✓	Observation
Weight		✓	N/A	Observation
Height		✓	N/A	Observation
History of present illness		✓	N/A	Encounter and Condition
Physical examination		✓	✓	Observation
Diagnosis		✓	✓	Condition
Investigation		N/A	✓	Observation, Procedure, and ImagingStudy
Drug name or dose (management in ASEAN MDS)		✓	✓	Medication and MedicationRequest
Procedure		N/A	✓	Procedure
**Staff signature**				
	Reception	✓	✓	Practitioner
	Doctor	✓	N/A	Practitioner
	MDS	✓	N/A	Practitioner
	Nurse	✓	N/A	Practitioner
	Drug	✓	N/A	Practitioner
	Examination	✓	N/A	Practitioner
	Data input	✓	✓	Practitioner
Memo		✓	✓	Observation

a WHO: World Health Organization.

b EMT: emergency medical team.

c MDS: minimum dataset.

d ASEAN: Association of Southeast Asian Nations.

e FHIR: Fast Healthcare Interoperability Resources.

f N/A: not applicable.

g ✓: match.

h ╳: not a match.

i BT: body temperature.

j PR: pulse rate.

k BP: blood pressure.

l RR: respiratory rate.

m O_2_ sat: oxygen saturation.

n GCS: Glasgow Coma Scale.

**Table 2.** Mapping results in the daily reporting form with tick boxes.

Variables		Match or not a match	FHIR^a^ resources
**MDS^b^ statistics**			
Age		╳^c^	N/A^d^
Sex		✓^e^	Patient, Observation
Health events		✓	Condition
**Procedure and Outcome**			
	Procedure	✓	Procedure
	Outcome	✓	Condition, Observation, and ServiceRequest
**Context**			
	Relation	╳	N/A
	Protection	✓	Condition
**Daily reporting form**			
**Team information**			
	Organization name	✓	Organization
	Team name	✓	Organization
	Type 1 mobile	✓	Organization
	Type 1 fixed	✓	Organization
	Type 2	✓	Organization
	Type 3	✓	Organization
	Specialized cell	✓	Organization
	Contact person(s) name(s)	✓	Organization
	Phone number	✓	Organization
	Email	✓	Organization
	Estimated date departure	╳	N/A
	Date of activity	✓	Location
	Time of reporting	✓	Encounter
**Location**			
	State	╳	N/A
	City	╳	N/A
	Village	╳	N/A
	Facility name	✓	Location
	Geo-tag (latitude)	✓	Location
	Geo-tag (longitude)	✓	Location
**Daily summary**			
	Total number of new consultation	✓	Encounter
	New admission	✓	Encounter
	Live birth	✓	Patient and Encounter
	Total bed capacity	✓	Location
	Empty inpatient bed (non-ICU^f^)	✓	Location
	Empty ICU	✓	Location
**Needs and Risks**			
**Immediate report**			
	Unexpected death	✓	Communication
	Notifiable disease	✓	Communication
	Protection issues #	✓	Communication
	Critical incident to EMT^g^ and/or community	✓	Communication
	Any other issue requiring immediate reporting	✓	Communication
**Community risks**			
	WASH^h^	✓	Communication
	Community or suspected over infectious disease	✓	Communication
	Environmental risk or exposure	✓	Communication
	Shelter or nonfood items	✓	Communication
	Food insecurity	✓	Communication
**Operational constrains**			
	Logistics or operational support	✓	Communication
	Supply	✓	Communication
	Human resources	✓	Communication
	Finance	✓	Communication
	Others	✓	Communication

a FHIR: Fast Healthcare Interoperability Resources.

b MDS: minimum dataset.

c ╳: not a match.

d N/A: not applicable.

e ✓: match.

f ICU: intensive care unit.

g EMT: emergency medical team.

h WASH: water, sanitation and hygiene.

**Table 3.** FHIR^a^ usability.

FHIR resources	WHO^b^ EMT^c^ MDS^d^	ASEAN^e^ EMT MDS	Daily reporting form
Organization	N/A^f^	✓^g^	✓
Location	N/A	✓	✓
Encounter	✓	✓	✓
Patient	✓	✓	✓
Observation	✓	✓	✓
Immunization	✓	✓	N/A
AllergyIntolerance	✓	✓	N/A
Condition	✓	✓	✓
MedicationStatement	✓	✓	N/A
MedicationRequest	✓	✓	N/A
Procedure	N/A	✓	✓
ServiceRequest	✓	✓	✓
Practitioner	✓	✓	N/A
ImagingStudy	N/A	✓	N/A
Communication	N/A	N/A	✓
Total, n	10	14	9

a FHIR: Fast Healthcare Interoperability Resources.

b WHO: World Health Organization.

c EMT: emergency medical team.

d MDS: minimum dataset.

e ASEAN: Association of Southeast Asian Nations.

f N/A: not applicable.

g ✓: use.

**Table 4.** Calculation of EMT^a^ MDS^b^ variables mapped to FHIR^c^ resources.

Form	Variables, n	Matching variables, n (%)
WHO^d^ EMT MDS	30	30 (100)
ASEAN^e^ EMT MDS	32	31 (97)
Daily reporting form	46	39 (85)

a EMT: emergency medical team.

b MDS: minimum dataset.

c FHIR: Fast Healthcare Interoperability Resources.

d WHO: World Health Organization.

e ASEAN: Association of Southeast Asian Nations.

### Gap Analysis

**Figure 2.**
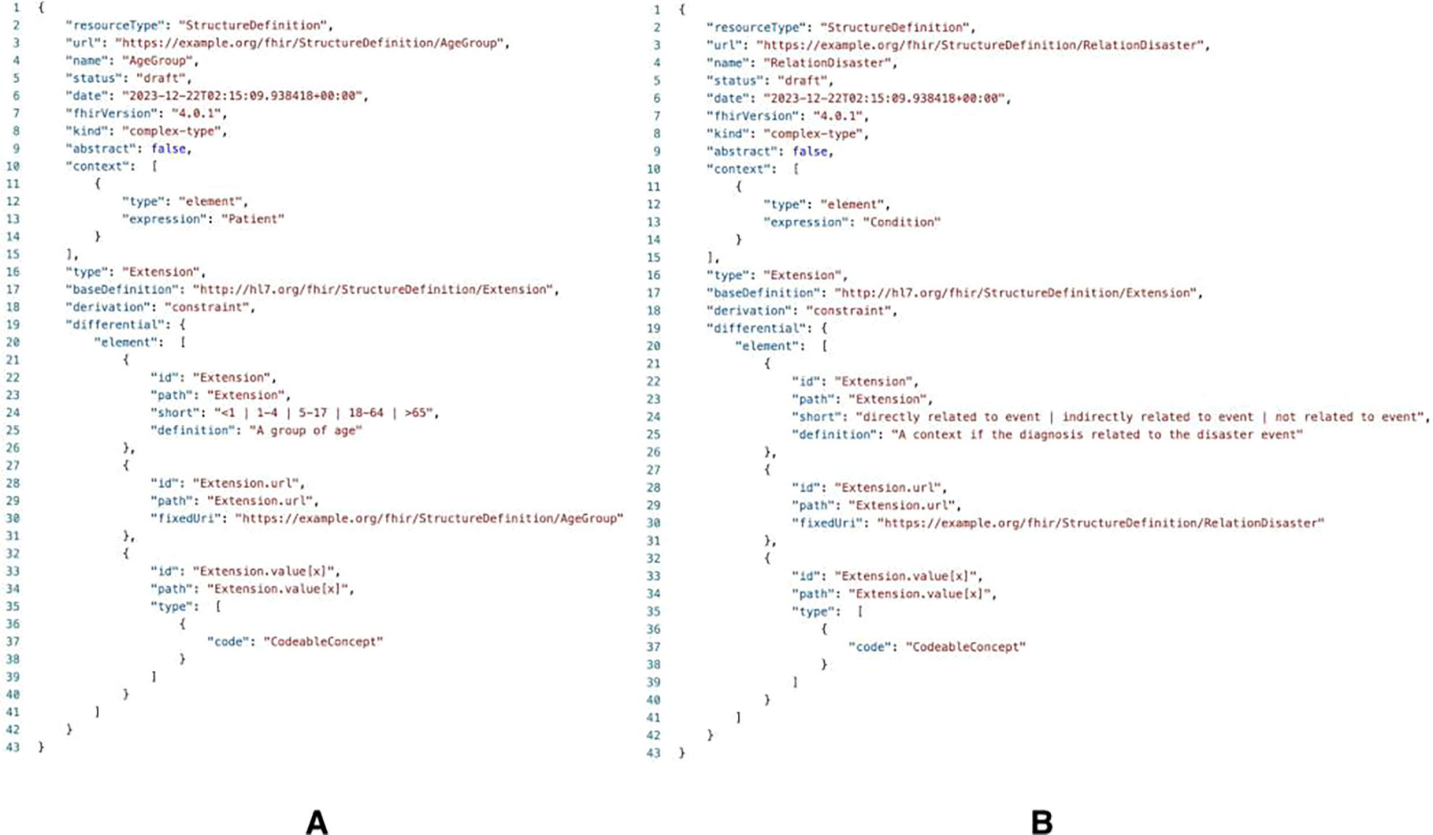
Extensions for (A) age and (B) relation data elements.

From the gap analysis, we found several data elements that did not match the FHIR resources. Most data came from the daily reporting form. The list is presented in Table 5 . We created extensions for the age, relationship, and estimated date departure elements for this problem. Age did not contain a data element in the FHIR resources. Because age is related to patients, we created an extension of the patient resource to define it. The JSON file is shown in Figure 2.

Relation status is defined as whether the patient’s condition is related to a disaster event. Thus, an extension of the condition resource is deemed appropriate to explain this condition. By definition, the estimated date of departure is when the team ends its service at specific locations. This allows the EMTCC to plan another EMT if necessary. Because this definition differs from the operational service hours in which the data element is available in the Location resource, we added an extension under the Organization resource to define the end period of the EMT service.

Sex in medical records uses a different value set from FHIR, divided into 3 groups: male, pregnant female, and nonpregnant female. Although sex is only divided into 2 groups, it adds pregnancy status to the female criteria. Since the pregnancy status already has a code in Logical Observation Identifiers Names and Codes (LOINC; “pregnancy status reported,” code 11449‐6), we bundled the Patient and Observation resources for this variable.

**Table 5.** Gap analysis results.

Data elements	Type	Form	Remarks	Possible FHIR^a^ resources
Age	String	ASEAN^b^ EMT^c^ MDS^d^ and daily reporting form	No exact definition in the data element of FHIR	Patient
State	Code	Daily reporting form	The data element available in ID core extension	Location
City	Code	Daily reporting form	The data element available in ID core extension	Location
Village	Code	Daily reporting form	The data element available in ID core extension	Location
Gender	Code	Daily reporting form	The definition implicate in 2 resources	Patient and Observation
Relation	Code	Daily reporting form	No exact definition in the data element of FHIR	Condition
Estimated date departure	Date	Daily reporting form	No exact definition in the data element of FHIR	Organization

a FHIR: Fast Healthcare Interoperability Resources.

b ASEAN: Association of Southeast Asian Nations.

c EMT: emergency medical team.

d MDS: minimum dataset.

### Validation and Implementation

**Figure 3.**
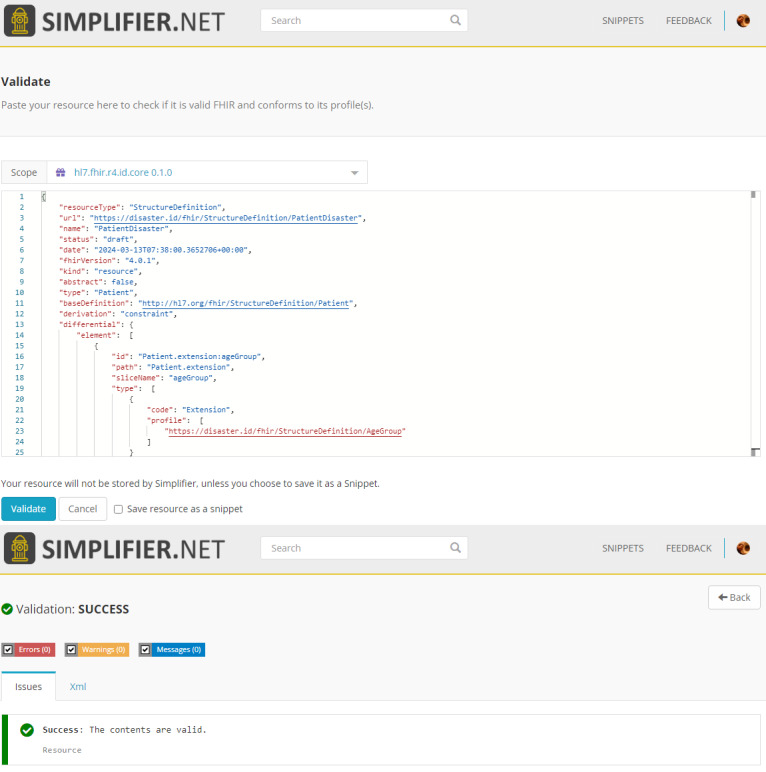
Validation results on SIMPLIFIER.NET.

We validated the resources for the FHIR ID core using SIMPLIFIER.NET. Example results are shown in Figure 3. The proposed disaster FHIR profile is implemented using the HAPI framework. We used the Insomnia application to perform requests on the FHIR REST server.

**Figure 4.**
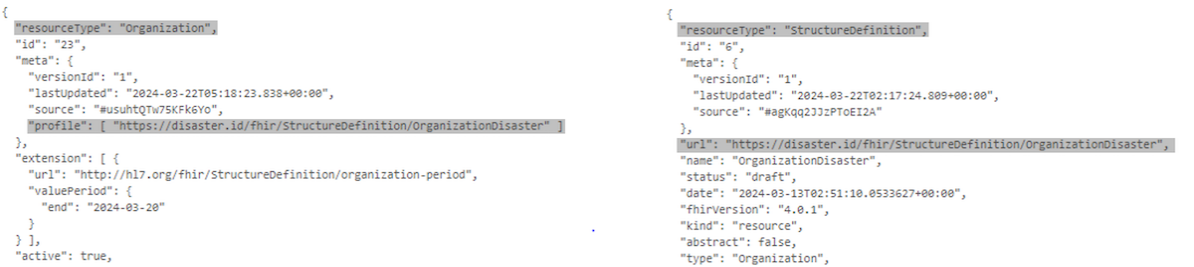
A JSON of the disaster profile was created based on HAPI. The profile mentioned (left) matches with the StructureDefinition resource (right).

We created several mock datasets based on the SATUSEHAT public example [29]. We managed to have samples for each resource. However, we added extensions that are not defined in the SATUSEHAT platform. Afterward, we validated the disaster profile using the HAPI FHIR server. We created a JSON file for each resource to implement the POST protocol. The POST protocol validates the request body and stores the resources in a database. We successfully validated 15 resources used in this profile (an example message is shown in Figure 4).

## Discussion

### Principal Findings

Data collection plays a significant role during disasters. With major calamities requiring support from international aid workers, implementing a standard data format can greatly facilitate the collection of health-related information. We chose the WHO and ASEAN EMT MDS forms to map onto the FHIR profiles. Although the EMT MDS is a standard developed by the WHO for data collection during disasters, the technical implementation designs for disaster situations in which data are transferred manually using an Excel spreadsheet [18]. As daily reports from various sources may be used by the MoH or EMTCC to make decisions, shifting from paper-based documentation to a digital process may increase the efficiency and effectiveness of collecting daily reports from various sources. In this study, we successfully created and validated the profile to the FHIR ID core profile using base resources. A FHIR profile has generally been created for specific use cases; however, few studies have performed disaster-related profiling. Several studies have created profiles related to disasters, such as COVID-19 [30,31].

To the best of our knowledge, this is the first study that has developed FHIR profiles for natural disasters. Our profiles may standardize health data collection, resulting in real-time actionable insights during disaster scenarios. For example, the interoperability facilitated by FHIR allows instant data sharing between disparate health systems, ensuring that international aid workers and medical facilities can access up-to-date patient records, treatment histories, and socioeconomic backgrounds. This seamless exchange of information is essential for coordinating effective and timely responses, significantly affecting survival rates and recovery outcomes.

Another advantage of using the FHIR format is that it optimizes the usability and quality of the health data. The EMT MDS form only records the number of disaster incidents. For example, if a patient visited twice in a day, 2 reports were generated [20]. Using FHIR, we can retrieve existing patient data from the database. The data entered during a disaster can be saved on the SATUSEHAT platform and reused when referring disaster survivors to nearby health care facilities [32]. By doing so, the quality and safety of patient care can be enhanced. These interoperability features have also enabled us to improve the efficiency of clinical data utilization during and after disasters. Ayaz et al [33] discussed the implementation of FHIR applications in their systematic literature review. Balch et al [34] defined the use of FHIR data standards in machine learning–enabled clinical information systems. Thus, using the data available on the SATUSEHAT platform would be helpful for researchers to perform studies related to disasters, such as identifying patterns of disease outbreaks and improving decision-making processes.

We mapped over 80% of the WHO and ASEAN EMT MDS medical record variables onto FHIR resources. Meanwhile, the variables that cannot be mapped to FHIR resource are mainly in the tick-box sections. Variables that did not have data elements, such as age and sex, were due to the characteristics of the EMT MDS form itself. The EMT MDS form was created to facilitate the collection of data and reports on the conditions occurring in the field [35]. This report form is an accumulation of the various daily report forms used by the EMTCC and MoH to provide care and treatment. Thus, age and sex were classified in a classification format to simplify grouping.

### Limitations

This study faced a notable challenge in developing a comprehensive value set for EMT MDS tick-box variables. FHIR uses a standard value set to define medical terminology, such as Systematized Nomenclature of Medicine–Clinical Terms (SNOMED-CT) or LOINC codes. We could not define the value options for health events and procedure variables because the value options from these variables were a group of several conditions. For example, health events have a “major extremity injury” option, which by definition indicates any upper- and lower-extremity injury requiring hospitalization and/or spinal or general anesthesia [36]. However, SNOMED-CT did not have codes for this category; it did have codes to define each of the upper- and lower-extremity injuries (the detailed list is available in Multimedia Appendix 1). Some studies have reported similar findings when mapping the values to SNOMED-CT or LOINC codes [37,38]. A previous study also mentioned that SNOMED-CT requires disaster codification adjustments [39]. One solution that has been done is to submit new codes to the SNOMED-CT or LOINC committee. However, this process is often prolonged without a guarantee of acceptance of the proposed code [40]. An alternative, more immediate solution we considered was to create a new value set that aligns with the WHO EMT MDS standards [41]. This approach aims to bridge the current gap in codification and facilitate more accurate data representation and analysis for disaster health management.

Finally, because this study was based on the concept of the mapping process, we did not perform a functionality test using real data. We used a mock-up dataset to test the POST and GET functions on the FHIR server. In the future, we will conduct tests using data from the SATUSEHAT platform by opening up opportunities for collaboration with the Indonesian MoH. In addition, future work should focus on FHIR-based applications using disaster profiles for health data collection during disaster events.

### Conclusions

This research aims to facilitate data collection using international standards, such as the WHO EMT MDS, and transfer it to the SATUSEHAT platform using FHIR as an interoperability standard. The proposed disaster profile was successfully implemented and validated using the FHIR server. This research will be beneficial for facilitating data collection using international standards during disasters and transferring it to a national platform for health care activity using FHIR as an interoperability standard.

## Supplementary material

10.2196/59651 Multimedia Appendix 1 Table of minimum dataset mapping to Systematized Nomenclature of Medicine–Clinical Terms (SNOMED-CT).

## Abbreviations

API: application programming interface
ARCH Project: Project for Strengthening the Association of Southeast Asian Nations Regional Capacity on Disaster Health Management
ASEAN: Association of Southeast Asian Nations
EMT: emergency medical team
EMTCC: Emergency Medical Team Coordination Cell
FHIR: Fast Healthcare Interoperability Resources
LOINC: Logical Observation Identifiers Names and Codes
MDS: minimum dataset
MoH: Ministry of Health
REST: representational state-transfer
SNOMED-CT: Systematized Nomenclature of Medicine–Clinical Terms
WHO: World Health Organization
